# Effect of* Curcuma xanthorrhiza* Supplementation on Systemic Lupus Erythematosus Patients with Hypovitamin D Which Were Given Vitamin D_3_ towards Disease Activity (SLEDAI), IL-6, and TGF-*β*1 Serum

**DOI:** 10.1155/2017/7687053

**Published:** 2017-12-28

**Authors:** C. Singgih Wahono, Cameleia Diah Setyorini, Handono Kalim, Nurdiana Nurdiana, Kusworini Handono

**Affiliations:** ^1^Rheumatology and Immunology Division, Department of Internal Medicine, Faculty of Medicine, Brawijaya University and Saiful Anwar General Hospital, Malang, Indonesia; ^2^Department of Internal Medicine, Faculty of Medicine, Brawijaya University and Saiful Anwar Hospital, Malang, Indonesia; ^3^Pharmacology Department, Faculty of Medicine, Brawijaya University, Malang, Indonesia; ^4^Clinical Pathology Department, Faculty of Medicine, Brawijaya University, Saiful Anwar General Hospital, Malang, Indonesia

## Abstract

**Background:**

Curcumin contained in* Curcuma xanthorrhiza* is an immunomodulator that has similar biological effect as vitamin D. Combination of curcumin and vitamin D_3_ is expected to work synergistically.

**Objective:**

To determine the effect of* Curcuma xanthorrhiza* supplementation on vitamin D_3_ administration to SLEDAI, IL-6, and TGF-*β*1 serum in SLE patients with hypovitamin D.

**Methods:**

This was a double-blind RCT conducted in Saiful Anwar Hospital, Malang, in January 2016–March 2017. Subjects were SLE active (SLEDAI > 3) with levels of 25(OH)D3 ≤ 30 ng/ml and divided into two groups: those receiving cholecalciferol 3 × 400 IU and placebo 3 × 1 tablets (group I) and those receiving 3 × 400 IU cholecalciferol and* Curcuma xanthorrhiza* 3 × 20 mg for 3 months (group II). SLEDAI, levels of vitamin D, IL-6, and TGF-*β*1 in serum were evaluated before and after the treatment.

**Results:**

There were no significant differences in SLEDAI reduction, decreased serum levels of IL-6, and increased levels of TGF-*β*1 serum among groups after the treatment. Decreased levels of serum IL-6 have a positive correlation with SLEDAI reduction.* Conclusion. Curcuma xanthorrhiza* supplementation on vitamin D_3_ had no effects on SLEDAI and serum levels of IL-6 and TGF-*β*1. This clinical trial is registered with NCT03155477.

## 1. Background

Systemic lupus erythematosus (SLE) is a chronic, systemic autoimmune inflammatory disease that can affect almost every organ of the human body and is characterized by excessive production of autoantibodies [1]. SLE disease activity is influenced by dendritic cell abnormalities, the balance of CD4 + cell subset (Th1, Th2, Th17, and Treg), and levels of proinflammatory and anti-inflammatory cytokines and their receptors and autoantibody production in the body [2, 3]. Moreover, some studies suggest that SLE patients with hypovitamin D also have more severe clinical and disease activity [4, 5]. Eighty-four percent of SLE patients in Indonesia had hypovitamin D (vitamin D levels (25(OH)D3) serum <30 ng/mL) [6]. This is because of photosensitivity in SLE patients so they will tend to avoid sun exposure, drugs, corticosteroid [7] and chloroquine [8], insufficient vitamin D intake, and anti-vitamin D antibodies [9]. There were increases of interleukin-6 (IL-6) in 41 SLE patients with hypovitamin D, resulting in dominance of IL-6 over transforming growth factor-*β*1 (TGF-*β*1) and Th17 cells differentiation rather than Treg cells [10].

Rifai et al. (2014) gave cholecalciferol 1200 IU/day or 30 *μ*g/day for 3 months in 39 SLE patients with hypovitamin D, and it showed an increase of serum vitamin D level (6.55 ± 1,27 ng/mL, *p* = 0,00) and significant improvement of Systemic Lupus Erythematosus Disease Activity Index (SLEDAI) (6,45 ± 3,07, *p* = 0,00) and Fatigue Severity Scale (FSS) (2,27 ± 0,73, *p* = 0,00) after supplementation [11]. The disease activity improvement is due to an extracellular function of vitamin D in regulating immune response by decreasing the response of Th1 and Th17 cells, suppressing dendritic cell maturation, decreasing proliferation and activating B-cell maturation, increasing Treg cell number, inducing IL-4 (Th2), and improving the function of NKT cells. Vitamin D can suppress IL-6 and increase TGF-*β*1 [10]. Although there was an increase in vitamin D levels to normal (30.44 ± 3.16) and improvement in disease activity/SLEDAI to 6.20 ± 2.67 after treatment, the disease activity was still moderate, whereas the goal of therapy in SLE patients is achieving remission (SLEDAI score is 0) or mild activity (SLEDAI score is 1–5), so an additional drug is needed that can improve the response of vitamin D_3_ therapy.

Curcumin is a polyphenol compound widely found in ginger plants—*Curcuma longa *and* Curcuma xanthorrhiza*—and has potential as an immunomodulator for the treatment of SLE [12]. Curcumin decreases the percentage of Th1, Th2, Th17 and increases the percentage of Treg cells. Curcumin regulates proinflammatory cytokines IL-1*β*, IL-6, IL-12, TNF-*α*, and IFN-*γ* [13]. Curcumin also works synergistically with vitamin D in regulating the immune system. Curcumin is a natural ligand for vitamin D receptor (VDR) [14] and it is recently also mentioned that VDR activation can also induce Treg cells and inhibit the activation of Th17 [15].

Pratama et al. (2015) found there were a Th17 decrease and an increase of Treg cells after supplementation of 200 mg/kg/day (equivalent to 1551 mg/70 kg in humans) curcumin in SLE mice [16]. Khajehdehi et al. (2012) supplementation of curcumin 22.1 mg, 3 times daily for 3 months, in relapse or refractory lupus nephritis patients significantly reduced proteinuria, hematuria, and systolic blood pressure without any adverse effects [17]. Nonetheless, the utilization of curcumin for SLE still has not been widely studied, including its combination with vitamin D in SLE treatment.

The synergistic nature of curcumin with vitamin D in regulating immune cells provides an opportunity for researchers to improve the response of vitamin D_3_ therapy, improve clinical symptoms, decrease disease progression, and reduce mortality rates in SLE patients with hypovitamin D by combining curcumin supplementation with vitamin D. Curcumin supplementation on vitamin D_3_ administration may affect the balance of TGF-*β*1/IL-6 cytokines at cellular level.

## 2. Methods

This was a double-blind randomized controlled trial conducted in Saiful Anwar Hospital, Malang, in January 2016–March 2017. This research was conducted after obtaining ethical approval from the Saiful Anwar Hospital, Malang.

The sample size was calculated by the formula for unpaired numerical analytic research with one-way hypothesis. The minimum sample size required in this study is 34. The subjects who fulfilled inclusion criteria, were willing to participate in the research, and signed the informed consent were 40 patients.

Research subjects were randomized using simple randomization. The subjects were divided into 2 groups: the group receiving 3 × 400 IU cholecalciferol and 3 × 1 tablet placebo (group I, *n* = 20) and the group receiving 3 × 400 IU and curcumin* (Curcuma xanthorrhiza)* 3 × 20 mg for 3 months (group II, *n* = 20). The dosage of vitamin D given in this study was based on previous study by Rifai et al. (2014), who successfully increased level of vitamin D into normal levels [11], while the dosage of curcumin was determined by a previous study by Khajehdehi et al. (2012) [17].

The inclusion criteria were patients diagnosed as SLE based on the criteria of American College of Rheumatology (ACR) 1997, active SLE (SLEDAI > 3), and 25(OH)D level < 30 ng/ml. Exclusion criteria were patients who were pregnant, taking supplements containing vitamin D and curcumin, had liver function disorders (AST/ALT levels > 2.5 times the upper normal limit), and had impaired renal function (GFR < 25 ml/min) and severe infections such as tuberculosis, pneumonia, or HIV.

Fifteen cc of venous blood samples were taken for complete blood tests, liver function (AST/ALT), renal function (urea/creatinine), vitamin D (25(OH)D), calcium, anti-dsDNA, C3, C4, IL-6, and serum TGF-*β*1. In examination of vitamin D levels Enzyme Immunoassay method was used (DiaSorin Inc., Stillwater, MN, USA), in examination of anti-dsDNA levels ELISA was used (Bioluminescenassay), in examination of C3, C4, IL-6, and serum TGF-*β*1 ELISA was used (Biolegend). Proteinuria was examined by using a urine spot sample with enzymatic-turbidimetric methods. SLE disease activity was assessed using SLEDAI score. Laboratory and SLEDAI examinations were performed at the beginning of the study and the end of the study, except for the serum calcium levels examined each month to determine the side effects of the drug.

Patients continue to receive the usual immunosuppressant drugs (corticosteroids, chloroquine, cyclophosphamide, mycophenolate mofetil, azathioprine, and cyclosporine), as well as calcium, antihypertensive drugs, and other routine medications. A physician in the Rheumatology outpatient clinic who is not a member of the research team in different examination rooms administers regular drugs, cholecalciferol,* Curcuma xanthorrhiza,* and placebo.

The results are presented in mean ± standard deviation, median (IQR 25–75%), and *n* (%). The homogeneity test of variance uses Levene. The Shapiro-Wilk test is used to determine the normality of the data, assuming the normality of the data is fulfilled if *p* > 0.05. Different tests after the treatment in both study groups use a nonpaired/Mann–Whitney test. The influence between variables is tested using Spearman/Pearson correlation test. Data analysis uses Statistical Package for the Social Sciences Software version 22 (SPSS Inc., Chicago, IL). Differences and correlations are said to be significant when the value of *p* is <0.05.

## 3. Results

### 3.1. Characteristics of Research Subjects

Subjects who participated in this study were 39 patients and were divided into 2 groups: twenty patients in vitamin D_3_ and placebo group and 19 patients in vitamin D_3_ and* Curcuma xanthorrhiza* group (1 patient in this group dropped out because she did not take the medication routinely).

Characteristic data in this study included age, duration of illness, initial clinical manifestations, medications, SLEDAI score, vitamin D, calcium, anti-dsDNA, C3, C4, IL-6, and serum TGF-*β*1 and protein-creatinine ratio (Tables 1 and 2). There were no significant differences between the two groups.

### 3.2. Effects of* Curcuma xanthorrhiza *Supplementation on Vitamin D_3_ Administration towards Vitamin D Level and SLEDAI in SLE Patients with Hypovitamin D

**Figure 1:**
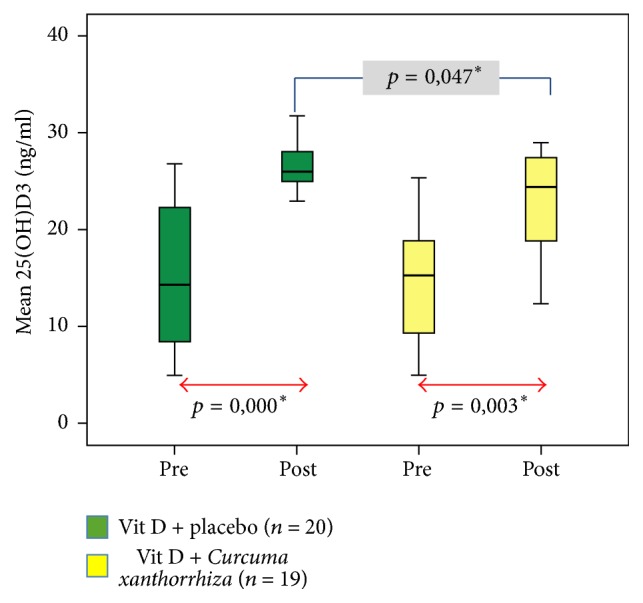
Effects of* Curcuma xanthorrhiza* supplementation on vitamin D_3_ administration towards vitamin D level. ^*∗*^There is a significant difference (*p* < 0.005).

**Figure 2:**
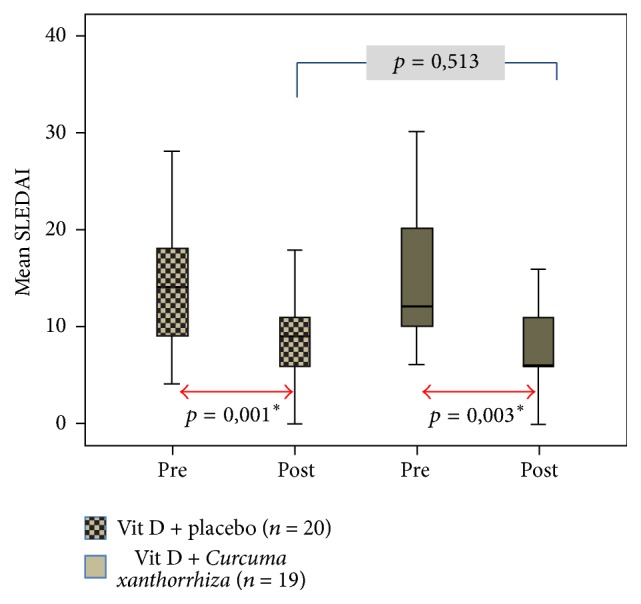
Effects of* Curcuma xanthorrhiza* supplementation on vitamin D_3_ administration towards SLEDAI. ^*∗*^There is a significant difference (*p* < 0.005).

Figure 1 shows a significant increase in serum vitamin D levels in each group after the treatment. In group I, the average of vitamin D level increased from 14.9 ± 7.5 ng/ml to 26.8 ± 3.7 ng/ml (*p* = 0,000), whereas, in group II, the average of vitamin D levels increased from 14.3 ± 6.5 ng/ml to 22.7 ± 5.4 ng/ml (*p* = 0.003). Although serum vitamin D levels differed significantly after the treatment, with higher levels of vitamin D in group I (*p* = 0.047), the delta of vitamin D levels (the difference between vitamin D levels after supplementation and before supplementation) did not differ significantly between the two groups (11.9 ± 6.2 ng/ml versus 8.4 ± 9.1, *p* = 0.166). Five patients (26.3%) experienced a decrease in serum vitamin D levels in group II. Levels of vitamin D serum after supplementation in this study are still insufficient.  Figure 2 shows a significant improvement of SLEDAI score in each group after treatment. In group I, the mean SLEDAI score decreased from 15.1 ± 7.6 to 9.1 ± 5.6 (*p* = 0.001), whereas, in group II, the mean SLEDAI score decreased from 14.8 ± 7.9 to 9.2 ± 7.4 (*p* = 0.003). SLEDAI score after treatment among groups did not differ significantly (*p* = 0.513). The delta of SLEDAI score reduction after treatment was not significantly different between the two groups (5.9 ± 5.3 versus 5.6 ± 6.6, *p* = 0.870). However, the activity of SLE disease in the study subjects after treatment was still moderate activity (SLEDAI score 6–10).

### 3.3. Effects of* Curcuma xanthorrhiza *Supplementation on Vitamin D_3_ Administration towards IL-6 and TGF-*β*1 Serum in SLE Patients with Hypovitamin D

Table 3 shows a marked decrease in serum IL-6 levels, elevation serum TGF-*β*1, and ratio TGF-*β*1/IL-6 levels in both groups. The delta of decreased serum IL-6 levels and elevated TGF-*β*1 levels did not differ significantly after the treatment for the two groups (Table 4). However, the delta ratio TGF-*β*1/IL-6 was significantly different among the two groups.

In group I, mean serum IL-6 levels decreased from 12.7 (6.6–18.1) pg/ml to 3.6 (2.6–4.8) pg/ml (*p* = 0.001), whereas, in group II, serum IL-6 levels decreased from 6.8 (4.7–15.4) pg/ml to 5.2 (3.2–6.4) (*p* = 0.013), respectively. Nevertheless, the mean difference (*p* = 0.061) and the amount of serum IL-6 levels decrease (*p* = 0.061) after the treatment was not found to be a significant difference. Several SLE patients in the vitamin D_3_ and* Curcuma xanthorrhiza* group experienced higher serum IL-6 levels (36.8%) more than in the vitamin D_3_ and placebo groups (15%), but it did not differ significantly.

In group I, the mean serum TGF-*β*1 level increased from 289.1 (2488.8–341.9) pg/ml to 355.9 (328.8–427.1) pg/ml (*p* = 0,000), whereas, in group II, mean serum TGF-*β*1 levels increased from 279.3 (249.4–313.1) pg/ml to 348.4 (292.1–4386.8) pg/ml (*p* = 0.011). The mean and elevated serum TGF-*β*1 levels after treatment in both groups did not have a significant difference (*p* = 0.261; *p* = 0.535). Several patients from the vitamin D_3_ and* Curcuma xanthorrhiza* group had a decrease of serum TGF-*β*1 levels after treatment (21.0%), whereas in the vitamin D_3_ and placebo group only one patient (5%) had a decrease of serum TGF-*β*1 levels after the treatment.

In group I, the mean serum TGF-*β*1/IL-6 ratio increased from 35.2 ± 22.3 to 78.6 ± 48.7 (*p* = 0.003), whereas, in group II, the ratio of TGF-*β*1/IL-6 serum average increased from 30.5 ± 18.1 to 115.6 ± 58.0 (*p* = 0.000). In group I, there was a significantly greater difference of TGF-*β*1/IL-6 ratio of 85.1 ± 58.1, compared to group II (43.4 ± 56.2) (*p* = 0.029). It means that vitamin D can increase TGF-*β*1 level and decrease the level of IL-6 greater than the combination of vitamin D and* Curcuma xanthorrhiza*. The greater the ratio of TGF-*β*1/IL-6 is, the more improved the activity of the disease will be.

### 3.4. Relationship between IL-6 and TGF-*β*1 Serum Changes with SLEDAI Score in SLE Patients with Hypovitamin D

**Figure 3:**
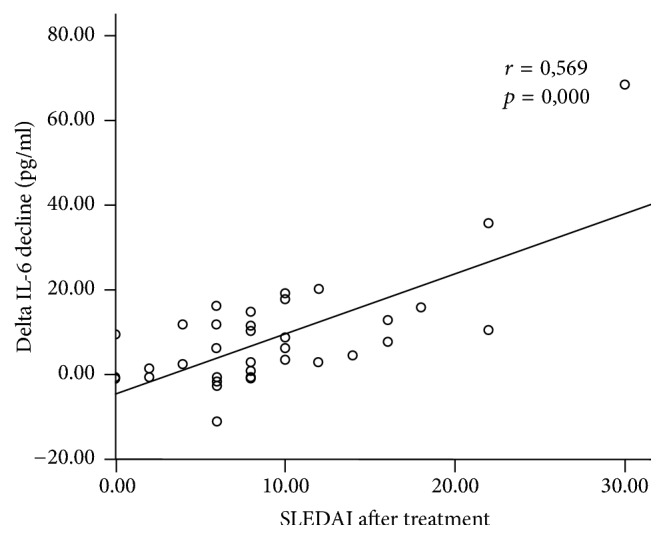
Correlation between SLEDAI and decline of IL-6 serum after supplementation.

**Figure 4:**
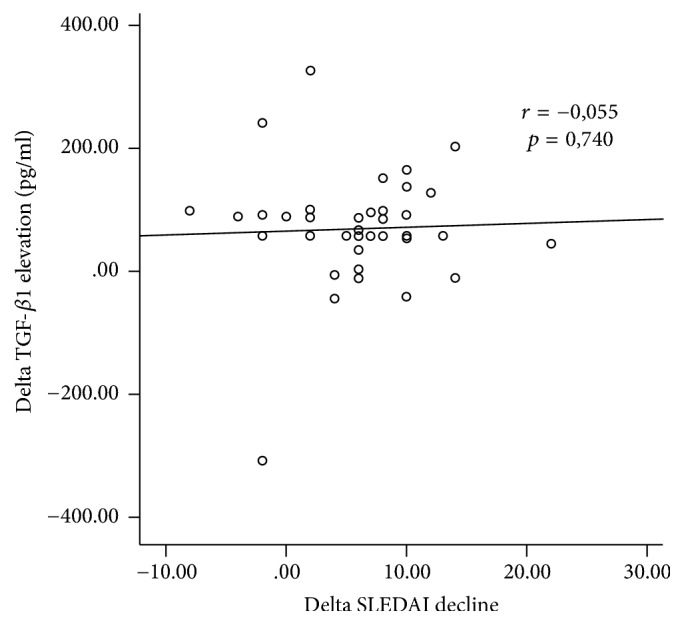
Correlation between SLEDAI and elevation of TGF-*β*1 serum after supplementation.

SLEDAI score after treatment positively correlated significantly with lower serum IL-6 levels (*r* = 0.569, *p* = 0.000). However, the strength of this correlation is moderate (Figure 3), while elevated serum TGF-*β*1 levels to SLE patient SLEDAI score had no significant correlation (*r* = 0.055, *p* = 0.740) (Figure 4).

## 4. Discussion

### 4.1. Increased Levels of Vitamin D

Supplementation of cholecalciferol 1200 IU either with placebo or with curcumin* (Curcuma xanthorrhiza)* 60 mg/day for 3 months was able to significantly increase serum 25(OH)D3 levels, but this increase has not yet resulted in normal levels of 25(OH)D3 serum (25(OH)D3 > 30 ng/ml). This may be due to the fact that patient's initial vitamin D levels were deficient (25(OH)D3 < 20 ng/ml) (65% in the vitamin D and placebo group, 73% in the vitamin D and* Curcuma xanthorrhiza* group). Therefore, to achieve normal levels of 25(OH)D3, supplementation should be given over a longer period of 4–6 months or with larger doses, but not exceeding the tolerable upper intake of 50 *μ*g/day equivalent to vitamin D_3_ 2000 IU [18]. Or we can give loading doses of vitamin D_3_ first in SLE patients with vitamin D deficiency. Van Groningen et al. (2010) mentioned that, in patients with vitamin D deficiency, loading dose cholecalciferol is required to achieve 25(OH)D3 75 nmol/l with the calculation of the required dose (IU) = 40 × (75-serum 25(OH)D3) × body weight [19]. NHS (2013) explains that the loading dose can be given up to 300,000 IU depending on the availability of vitamin D and given for 6–10 weeks in divided doses [20].

The average increase level of 25(OH)D3 in both groups was approximately 8.4–11.4 ng/ml but slightly lower in the vitamin D_3_ and* Curcuma xanthorrhiza* group. This is similar to previous research that the administration of cholecalciferol 1000 IU/day for 3-4 months can increase the levels of 25(OH)D about 10 ng/ml 25(OH)D [21], so that if supplementation cholecalciferol 1200 IU/day is given, it will increase serum 25(OH)D by 12 ng/ml. Although there were significant differences in serum 25(OH)D3 levels after the treatment in both groups, the ability to increase serum 25(OH)D3 levels did not differ significantly.

In the group given vitamin D_3_ and* Curcuma xanthorrhiza*, some patients (26%) experienced a decrease in serum 25(OH)D3 levels, whereas in the vitamin D_3_ and placebo groups none of them had a decrease of 25(OH)D serum levels. In the group of patients who had a decrease of serum 25(OH)D3 levels, all of them had LES with mucocutaneous manifestations and used methylprednisolone and chloroquine. Thus, a decrease in serum 25(OH)D3 levels may be due to the fact that the patient tends to avoid direct sun exposure either by limiting day-to-day activity, by applying sunscreen, or by using closed clothing to avoid flares due to photosensitivity.

The use of corticosteroids in the treatment of SLE also causes vitamin D deficiency because corticosteroids accelerate calcidiol catabolism (25(OH)_2_D_3_) into calcitriol (1,25(OH)_2_D_3_) [7]. Chloroquine inhibits the conversion of calcidiol (25(OH)_2_D_3_) into calcitriol (1,25(OH)_2_D_3_) [8]. In addition, it is also reported in 4% of SLE patients that there are antibodies of vitamin D [9], which are likely to affect vitamin D clearance, so even though vitamin D has been given in sufficient doses of 400–800 IU/day, the patient will remain in the status of insufficiency. Until now, there has been no reported absorption disorder, metabolism, or vitamin D clearance by curcumin.

### 4.2. Decline of SLEDAI Score

SLEDAI scores in both groups decreased significantly after treatment. This is in accordance with Rifai et al. research in 2013 [10]. Abou-Raya et al. (2013) also mentioned that supplementation of cholecalciferol 2000 IU/day for 12 months to SLE patients who had vitamin D levels < 30 ng/ml is able to improve disease activity significantly [22]. Similarly, Petri et al. (2013) explained that, in SLE patients with levels of 25(OH)D < 40 ng/ml, there was an improvement in disease activity after supplementation of vitamin D_2_ 50,000 IU/week and calcium twice daily [23]. However, it was said that the clinical importance of vitamin D was moderate.

The improvement of SLEDAI scores was higher in the vitamin D_3_ and placebo groups (SLEDAI 5.9 ± 5.3) compared to the vitamin D_3_ and curcumin group (SLEDAI 5.6 ± 6.6), although it was not significant. The improvement of SLEDAI score in vitamin D_3_ and placebo group was relatively the same compared to previous research conducted by Rifai et al. (2013), which in their previous study obtained a decrease in SLEDAI score on vitamin D_3_ administration of 6.4 ± 3.1. Some patients did not improve the SLEDAI score in both groups.

From this study, we can conclude that the ability of vitamin D to decrease the score of SLEDAI ranged from 5 to 6. Immunosuppressant is still required, but at least the required dosage becomes smaller and may reduce side effects.

The SLEDAI score after the treatment of both groups still showed moderate activity (SLEDAI score 6–10). This may be due to 25(OH)D serum levels which still do not sufficiently achieve (25(OH)D > 30 ng/ml) level. However, in Rifai et al.'s study (2013), although serum 25(OH)D levels have reached normal, SLEDAI scores have not yet achieved remission (SLEDAI score = 0) or mild activity (SLEDAI score 1–5). Disease activity in SLE is influenced by many factors, not only vitamin D status, but also environmental and biological factors [24]. However, improvement of vitamin D levels can at least help in lowering SLE disease activity, resulting in lower mortality and morbidity.

### 4.3. Decline of IL-6 Serum Levels and Its Relationship with SLEDAI

Serum IL-6 levels in both groups before the treatment were high (normal value < 6 pg/ml) [25]. The serum IL-6 levels were variable, and levels were higher depending on SLE manifestations such as lupus nephritis. Serum and urinary IL-6 levels elevated in active SLE patients [26].

Serum IL-6 levels decreased significantly in both groups after treatment. The decrease of IL-6 is due to the work of vitamin D_3_ and curcumin (*Curcuma xanthorrhiza* active ingredient) capable of lowering serum IL-6 levels by inhibiting IL-6 production by blocking p38MKP5 [27, 28].

Although vitamin D and curcumin reduced IL-6 levels, there was no significant difference in serum IL-6 levels of serum levels after the treatment in both groups. This may be caused by the fact that the curcumin dosage used was too small or the dose was adequate but the bioavailability was low, or probably due to the competition of curcumin against vitamin D in its bond to VDR so it does not produce a synergistic response [15].

Bioavailability of curcumin administered per oral route is low, with rapid metabolism and elimination [29]. Curcumin contained in* Curcuma xanthorrhiza* is only about 1-2%, compared to curcumin obtained from* Curcuma longa* ranging from 3 to 8% [30].

Bartik et al. (2010) explained that curcumin concentration 10^−4^ M was able to compete against ligand 1,25D tied with VDR as much as 50%. It was concluded that curcumin did not potentiate the action of 1,25D-VDR [15]. The combination of 10^−5^ M curcumin and 1,25D 10^−8^ M increased the expression of vitamin D responsive element (VDRE), RXRE, or GRE 2.16 times more than on cells that are given only 1.25D 10^−8^ M [15].

Although vitamin D and curcumin are able to bind VDRs, the ability of curcumin to bind VDR to alternative pocket (VDR-AP) is greater, whereas vitamin D is more likely to bind VDR in the genomic pocket (VDR-GP) [31, 32]. VDR activation by curcumin requires higher concentrations (10^−6^ to 10^−5^ M curcumin plasma concentrations, or the equivalent of 8 grams of curcumin per day) compared to VDR activation by 1,25D ligand so it can be concluded that curcumin is a weaker VDR ligand. Therefore, to improve the bioavailability of curcumin, it is necessary to prepare curcumin dosage forms which are added to certain substances such as piperine or converted into nanoparticles [33].

The anti-inflammatory effect of curcumin results from the activation of peroxisome proliferator-activated receptor-*γ* (PPAR-*γ*) [34], but the concept that whether curcumin is a ligand of PPAR-*γ* is controversial. Narala et al. (2009) have conducted a study that curcumin is a ligand of PPAR-*γ* [35]. Thus, curcumin anti-inflammatory effects via PPAR-*γ* are probably indirect or via other mechanisms.

The magnitude of the decrease in interleukin-6 levels had an effect on the SLEDAI score after the treatment. The higher serum IL-6 levels of SLE patients will have more severe disease activity [36]. Therefore, we hope that administering vitamin D and* Curcuma xanthorrhiza* may decrease IL-6 greater than vitamin D only and improves lupus disease activity.

### 4.4. Increased Levels of Serum TGF-*β*1 and Its Correlation with SLEDAI

The more dominant cytokines in the SLE were IL-6 and IL-23, whereas the levels of TGF-*β* significantly decreased [37]. Serum TGF-*β*1 levels had significant increases between the two groups, but there was no significant difference between serum TGF-*β*1 levels and magnitude elevated serum TGF-*β*1 levels after the treatment. This is because the work of curcumin inhibits TGF-*β*1, in contrast to vitamin D properties against TGF-*β*1. However, because the dose of curcumin used in this study is small, the effects of curcumin seem to be excluded by vitamin D.

Until now, no studies have attempted to investigate the effect of curcumin on TGF-*β*1 in SLE patients. However, curcumin at certain doses and times can inhibit TGF-*β*1 activity in regulating MMP-9 and activation of SMAD-2, ERK1/2, and p38 in breast cancer cells [38].

Whether the mechanism of vitamin D will increase levels of TGF-*β* is not clear yet [10]. In this study, TGF-*β*1 levels did not correlate with SLEDAI score.

### 4.5. Limitations

In this study, there were different SLE manifestation, disease activity, and varying degree of vitamin D levels. We did not divide subjects into 3 groups, so that we did not know the effect of curcumin and placebo supplementation towards the SLEDAI score and cytokine levels in SLE patients with hypovitamin D. Also, curcumin plasma concentration was not measured at the study.

## 5. Conclusion and Suggestion

### 5.1. Conclusion

*Curcuma xanthorrhiza* supplementation on vitamin D_3_ in SLE patients with hypovitamin D did not have different effects on levels of SLEDAI, IL-6, and TGF-*β*1 in serum. Decreased levels of serum IL-6 have moderate correlation with a decrease in SLEDAI score.

### 5.2. Suggestion

Preliminary research is needed to assess the amount of plasma concentration of curcumin in the preparation of curcumin to be used. It is also necessary to do research using curcumin with varying doses and formulas with high bioavailability. And research needs to be done with three different groups: curcumin and placebo groups, vitamin D and placebo groups, and a combination of vitamin D and curcumin.

## Figures and Tables

**Table 1:** Baseline characteristics of subjects.

Characteristics	Vitamin D_3_ + placebo (*n* = 20)	Vitamin D_3_ + *Curcuma xanthorrhiza* (*n* = 19)	*p* value
Age (y.o.) (mean ± SD)	30.3 ± 10.0	27.9 ± 7.9	0.415
Illness duration (years) (median; IQR)	2.25 (IQR: 1.0–4.0)	2.0 (IQR: 1.0–4.0)	0.937
Body mass index (kg/m^2^) (median; IQR)	20.8 (IQR: 16.4–27.3)	20.8 (IQR: 17.1–28.3)	0.915
SLEDAI (mean ± SD)	15.1 ± 7.6	15.2 ± 7.4	0.879
*Early manifestation (%)*			
Mucocutan	17 (43.6%)	13 (33.3%)	0.219
Arthritis	9 (23.1%)	7 (17.9%)	0.605
Nephritis (proteinuria, hematuria, pyuria, cylindruria)	4 (10.3%)	8 (20.5%)	0.135
Hematology (AIHA, leucopenia, thrombocytopenia)	2 (5.1%)	3 (7.7%)	0.589
Vasculitis	1 (2.6%)	1 (2.6%)	0.970
Serositis	1 (2.6%)	1 (2.6%)	0.970
Cerebral	3 (7.7%)	4 (10.3%)	0.622
*Medications (%)*			
(a) Non-Immunosuppressants			
(i) Methylprednisolone (<8 mg/day)	19 (48.7%)	19 (48.7%)	0.323
(ii) Calcium Carbonate	19 (48.7%)	15 (38.5%)	0.134
(b) Immunosuppressants			
(i) Chloroquine	10 (25.6%)	7 (17.9%)	0.408
(ii) Cyclosporine	2 (5.1%)	0 (0%)	0.157
(iii) Cyclophosphamide	1 (2.6%)	5 (12.8%)	0.065
(iv) Azathioprine	10 (25.6%)	14 (35.9%)	0.129
(v) Mofetil mycophenolate	0 (0%)	0 (0%)	—

IQR: interquartile.

**Table 2:** Laboratory characteristics of research subjects.

Characteristics	Vitamin D_3_ + placebo (*n* = 20)	Vitamin D_3_ + *Curcuma xanthorrhiza* (*n* = 19)	*p* value
Hb (g/dL) (mean ± SD)	11.2 ± 1.6	11.9 ± 1.26	0.122
Total Lymphocytes Count (TLC) (mean ± SD) (…/mm^3^)	1321.5 ± 608.2	1027.3 ± 384.4	0.081
Vitamin D (ng/ml) (mean ± SD)	14.9 ± 7.5	14.3 ± 6.5	0.779
Calcium (mg/dl) (mean ± SD)	8.9 ± 0.6	8.8 ± 0.4	0.630
Anti-dsDNA (IU/ml) (median; IQR)	43.7 (IQR: 22.7–207.0)	56.2 (IQR: 21.9–172.7)	0.903
AST (U/L) (mean ± SD)	25.4 ± 3.6	27.3 ± 3.3	0.641
ALT (U/L) (mean ± SD)	25.4 ± 4.6	25.2 ± 3.6	0.426
Urea (mg/dl) (mean ± SD)	28.4 ± 22.5	26.9 ± 10.7	0.491
Creatinine (mg/dl) (mean ± SD)	0.6 ± 0.2	0.8 ± 0.5	0.623
Protein-creatinine ratio (mg/g)	655 (IQR: 197.5–1637.5)	400 (IQR: 30.0–75.0)	
ESR (mm/jam) (mean ± SD)	41.6 ± 23.3	49.9 ± 9	0.235
C3 (ug/ml) (median; IQR)	688.5 (IQR: 623.2–776.1)	652.3 (IQR: 592.8–870.1)	0.643
C4 (mg/ml) (median; IQR)	0.2 (IQR: 0.1–0.3)	0.2 (IQR: 0.1–0.3)	0.908
IL-6 (pg/ml) (median; IQR)	11.1 (IQR: 6.6–18.1)	6.8 (IQR: 4.7–15.5)	0.396
TGF-*β*1 (pg/ml) (median; IQR)	286.6 (IQR: 248.8–341.9)	279.3 (IQR: 249.3–313.1)	0.627
Ratio TGF-*β*1/IL-6 (mean ± SD)	35.2 ± 22.3	30.5 ± 18.1	0.469

IQR: interquartile.

**Table 3:** Effects of *C. xanthorrhiza *supplementation on vitamin D_3_ administration towards cytokine levels.

Variable	Vitamin D_3_ + placebo (*n* = 20)			Vitamin D_3_ + *Curcuma xanthorrhiza* (*n* = 19)			*p* ^*∗*^
	Pre	Post	*p* value	Pre	Post	*p* value	
IL-6 (pg/ml)	12.7	3.6	0.001^*∗*^	6.8	5.2	0.013^*∗*^	0.061
	(IQR: 6.6–18,1)	(IQR: 2.6–4.8)		(IQR: 4.7–15.4)	(IQR: 3.2–6.4)		
TGF-*β*1 (pg/ml)	289.9	355.9	0.000^*∗*^	279.3	348.4	0.011^*∗*^	0.261
	(IQR: 248.8–341.9)	(IQR: 328.8–427.1)		(IQR: 249.4–313.1)	(IQR: 292.1–4386.8)		
TGF-*β*1/IL-6 (pg/ml)	35.2 ± 22.3	78.6 ± 48.7	0.003^*∗*^	30.5 ± 18.1	115.6 ± 58.0	0.000^*∗*^	0.038^*∗*^

*p* ^*∗*^ = differential test of the effects of *Curcuma xanthorrhiza* supplementation after treatment in both groups, expressed by *p*. ^*∗*^There is a significant difference (*p* < 0.005); IQR: interquartile.

**Table 4:** Effects of *C. xanthorrhiza* supplementation on vitamin D_3_ administration towards cytokine changes.

Variable	Vitamin D_3_ + placebo (*n* = 20)	Vitamin D_3_ + *Curcuma xanthorrhiza* (*n* = 19)	*p* value
Δ IL-6 (pg/ml)	8.4 (IQR: 2.6–13.1)	2.9 (IQR: −0.45–11.85)	0.061
Δ TGF-*β*1 (pg/ml)	71.3 (IQR: 57.8–93.5)	57.9 (IQR: −1.0–100.0)	0.535
Δ TGF-*β*1/IL-6	85.1 ± 58.1	43.4 ± 56.2	0.029^*∗*^

^*∗*^There is a significant difference (*p* < 0.005); ΔIL-6: IL-6 before treatment/IL-6 after treatment; ΔTGF-*β*1: TGF-*β*1 after treatment/TGF-*β*1 before treatment; ΔTGF-*β*1/IL-6: ratio TGF-*β*1/IL-6 before treatment to ratio TGF-*β*1/IL-6 after treatment.
